# Vestiges of Adaptation to the Mesophilic Environment in the Genome of *Tepiditoga spiralis* gen. nov., sp. nov.

**DOI:** 10.1264/jsme2.ME20046

**Published:** 2020-09-18

**Authors:** Koji Mori, Kenta Sakurai, Akira Hosoyama, Takeshi Kakegawa, Satoshi Hanada

**Affiliations:** 1 NITE Biological Resource Center (NBRC), National Institute of Technology and Evaluation (NITE), 2–5–8 Kazusakamatari, Kisarazu, Chiba 292–0818, Japan; 2 Tohoku University, Sendai, Miyagi 980–8578, Japan; 3 Bioproduction Research Institute, National Institute of Advanced Industrial Science and Technology (AIST), 1–1–1 Higashi, Tsukuba, Ibaraki 305–8566, Japan; 4 Graduate School of Science and Engineering, Tokyo Metropolitan University, 1–1 Minami-Osawa, Hachioji, Tokyo 192–0397 Japan

**Keywords:** Thermotogae, Tepiditoga spiralis, Mesophilic environment

## Abstract

A novel anaerobic heterotrophic strain, designated strain sy52^T^, was isolated from a hydrothermal chimney at Suiyo Seamount in the Pacific Ocean. A 16S rRNA gene analysis revealed that the strain belonged to the family *Petrotogaceae* in the phylum *Thermotogae*. The strain was mesophilic with optimum growth at 48°C and the phylum primarily comprised hyperthermophiles and thermophiles. Strain sy52^T^ possessed unique genomic characteristics, such as an extremely low G+C content and 6 copies of rRNA operons. Genomic analyses of strain sy52^T^ revealed that amino acid usage in the predicted proteins resulted from adjustments to mesophilic environments. Genomic features also indicated independent adaptions to the mesophilic environment of strain sy52^T^ and *Mesotoga* species, which belong to the mesophilic lineage in the phylum *Thermotogae*. Based on phenotypic and phylogenetic evidence, strain sy52^T^ is considered to represent a novel genus and species in the family *Petrotogaceae* with the proposed name *Tepiditoga spiralis* gen. nov., sp. nov.

Growth temperature is one of the important physiological features for characterizing bacteria and archaea, and psychrophiles, mesophiles, thermophiles and hyperthermophiles are categorized based on them. An optimum growth temperature of 45°C is typically the boundary that separates mesophiles and thermophiles, but may vary (Madigan *et al.*, 2002; Wagner and Wiegel, 2008; Taylor and Vaisman, 2010). At the molecular level, growth temperature has been shown to correlate with genome and protein properties (Zheng and Wu, 2010).

Bacteria of the *Thermotogae* lineage primarily comprise hyperthermophiles and thermophiles isolated from high-temperature environments, and the phylum is phylogenetically placed in a deep-branched position in the domain Bacteria. Thirteen genera have been described since *Thermotoga maritima* was initially discovered in geothermally heated marine sediments (Huber *et al.*, 1986), and Bhandari and Gupta recently categorized the phylum into 4 orders and 5 families using genome data (Bhandari and Gupta, 2014). They primarily grow by fermentation under strictly anaerobic and thermophilic conditions and have a characteristic outer sheath-like structure called a ‘toga’. These genomic features were found to be unique, and detailed analyses revealed lateral gene transfer from diverse lineages of both Bacteria and Archaea as well as the genomic machinery of adaptation to high-temperature environments (Nelson *et al.*, 1999; Zhaxybayeva *et al.*, 2009; Bhandari and Gupta, 2014).

In addition to hyperthermophilic and thermophilic species, previous studies predicted the presence of mesophiles known as “mesotoga” in the phylum based on examinations of 16S rRNA genes from mesophilic environments and enrichments (Chouari *et al.*, 2005; Nesbo *et al.*, 2006; Berlendis *et al.*, 2010; Nesbo *et al.*, 2010). “*Mesotoga sulfurireducens*” PhosAc3 was initially isolated as a mesophilic bacterium belonging to the phylum; however, a complete description of the strain is not yet available (Ben Hania *et al.*, 2011; 2015). *M. prima* MesG1.Ag.4.2^T^ isolated from a marine sediment was the first strain to have its characteristics formally described and grows optimally at 37°C (Nesbo *et al.*, 2012). *Mesotoga infera* VNs100^T^ was also retrieved from anoxic water in a deep aquifer, and the temperature range for growth was 30–50°C with an optimum at 45°C (Ben Hania *et al.*, 2013). Species belonging to the genus *Geotoga* grow under relatively mesophilic conditions, and the optimum growth temperatures of *Geotoga petraea* T5^T^ and *Geotoga subterranea* CC-1^T^ were 50 and 45°C, respectively (Davey *et al.*, 1993). Except for the genera *Mesotoga* and *Geotoga*, bacteria of the *Thermotogae* lineage generally comprise hyperthermophiles and thermophiles, with an optimum growth temperature of higher than 55°C.

Previous analyses of the G+C content of 16S rRNA genes and the amino acid composition of protein-coding genes suggested that thermophilic features are systematically original characteristics of bacteria of the *Thermotogae* lineage (Zhaxybayeva *et al.*, 2009), and ‘mesotoga’ may have adapted to mesophilic environments. The genome of *M. prima* MesG1.Ag.4.2^T^ was found to be markedly larger than that of other bacteria of the *Thermotogae* lineage, and 32% of predicted protein-coding genes were shown to be acquired by lateral gene transfer (Zhaxybayeva *et al.*, 2012). Based on these findings, Zhaxybayeva *et al.* suggested that the genomic features of *M. prima* were indicative of its adaption to a new lifestyle, such as a mesophilic environment.

A novel mesophilic anaerobic heterotroph, designated strain sy52^T^ and belonging to the phylum *Thermotogae*, was recently isolated from a deep-sea hydrothermal field. The present study focused on physiological and genomic analyses and discusses mesophilic adaptations by this strain. In addition, based on phenotypic characteristics as well as phylogenetic analyses, a novel taxon is proposed for the isolate with the name *Tepiditoga spiralis* gen. nov., sp. nov.

## Materials and Methods

### Sample collection, enrichment, and isolation

The sample for enrichment and isolation was collected from a deep-sea hydrothermal vent chimney in Suiyo Seamount, the Izu-Bonin Arc, the western Pacific Ocean by DSV *Shinkai6500* during the YK11-06 scientific cruise aboard the R/V *Yokosuka* (JAMSTEC, Yokosuka, Kanagawa, Japan) in August 2012. The region has a submarine caldera with numerous hydrothermal vents at a depth of 1,390‍ ‍m (Glasby *et al.*, 2000). Chips of an active chimney were selected for enrichment and were immediately inoculated on board.

The medium under a N_2_/CO_2_ (80:20‍ ‍[v/v]) atmosphere was added to a vial sealed with a butyl rubber stopper and aluminum cap for enrichment, and isolation comprised (L^–1^) 0.6‍ ‍g KH_2_PO_4_, 0.1‍ ‍g K_2_HPO_4_, 0.75‍ ‍g MgCl_2_·6H_2_O, 0.15‍ ‍g CaCl_2_·2H_2_O, 0.3‍ ‍g NH_4_Cl, 30‍ ‍g NaCl, 0.3‍ ‍g Na_2_SO_4_, 1.6‍ ‍g Na_2_S_2_O_3_, 3‍ ‍g Bacto Yeast Extract (Difco), 2‍ ‍mL trace element solution (Mori and Suzuki, 2008), 2‍ ‍mL vitamin solution (Mori and Suzuki, 2008), 1‍ ‍mg resazurin, 1‍ ‍g Na_2_CO_3_, and 0.5‍ ‍g Na_2_S·9H_2_O. After mixing ingredients, except for the vitamin solution, Na_2_CO_3_, and Na_2_S·9H_2_O, the medium was autoclaved under a N_2_/CO_2_ atmosphere. The vitamin and Na_2_CO_3_ solutions were sterilized with filtration. Na_2_S·9H_2_O solution autoclaved separately was then added to the medium. Anaerobic bacteria were cultivated at various temperatures for enrichment; after 1‍ ‍week of cultivation, bacterial growth was confirmed at 30°C. Regarding single strain isolation, colonies were allowed to form on medium solidified with 1.5% (w/v) agar (Difco Agar Noble) in vials for approximately 2 months. After a second purification step with the same solid medium, a pure culture of strain sy52^T^ was obtained.

### Physiological characterization

Cell morphology was routinely observed using phase-contrast microscopy (model AX-70; Olympus). Optical density (A660) was measured with a spectrophotometer (model U-2800; Hitachi). A direct cell count was performed under a fluorescent microscope by 4',6-diamidino-2-phenylindole (DAPI) staining on a polycarbonate membrane filter (K020N025A; Advantec). The concentrations of sulfate, thiosulfate, and nitrate were assessed by HPLC (model 2695 with conductivity detector model 432 and an IC-Pac Anion column; Waters) (Mori *et al.*, 2008).

The following substrates were examined as the sole energy and carbon sources: 10‍ ‍mM D-glucose, 10‍ ‍mM D-fructose, 10‍ ‍mM D-mannose, 10‍ ‍mM D-galactose, 10‍ ‍mM maltose, 10‍ ‍mM lactose, 10‍ ‍mM D-trehalose, 10‍ ‍mM sucrose, 10‍ ‍mM D-cellobiose, 10‍ ‍mM D-raffinose, 10‍ ‍mM D-arabinose, 10‍ ‍mM L-rhamnose, 10‍ ‍mM D-xylose, 10‍ ‍mM D-ribose, 10‍ ‍mM ribitol, 10‍ ‍mM D-mannitol, 10‍ ‍mM D-sorbitol, 20‍ ‍mM glycerol, 20‍ ‍mM citrate, 20‍ ‍mM pyruvate, 20‍ ‍mM succinate, 20‍ ‍mM malate, 20‍ ‍mM L-glutamate, 20‍ ‍mM butyrate, 20‍ ‍mM lactate, 20‍ ‍mM propionate, 5‍ ‍g L^–1^ starch, 1‍ ‍g L^–1^ yeast extract, 1‍ ‍g L^–1^ polypeptone, and 1‍ ‍g L^–1^ casamino acids. The substrate utilization test was also performed in the presence of 0.2‍ ‍g L^–1^ yeast extract. The utilization of the following electron acceptors was evaluated in the presence of 3‍ ‍g L^–1^ yeast extract as the substrate: 10‍ ‍mM thiosulfate, 10‍ ‍mM sulfate, 2 and 5‍ ‍mM sulfite, 5‍ ‍g L^–1^ elemental sulfur, 10‍ ‍mM fumarate, 10‍ ‍mM nitrate, 2 and 5‍ ‍mM nitrite, and 2 and 5% (v/v) oxygen. H_2_S production from sulfur compounds as electron acceptors was confirmed by FeS precipitation after the addition of one drop of 0.1 M FeSO_4_ solution to cultures in media without sulfide as the reducing agent. The effects of temperature, initial pH, and NaCl concentrations on growth in the presence of 3‍ ‍g L^–1^ yeast extract and 10‍ ‍mM thiosulfate were assessed by examining the time course of optical density changes with a temperature gradient incubator (model TN-2612; Advantec). The initial pH of the medium was adjusted by adding Na_2_CO_3_ or HCl solution.

Cellular fatty acids were methylated using a 5% HCl/methanol solution (Sasser, 1990) and analyzed by the MIDI microbial identification system and GC-MS (gas chromatograph model GC-2010; gas chromatograph mass spectrometer model GCMS-QP2010Plus; Shimadzu).

### Genome sequencing and analyses

Genomic DNA was extracted using the EZ1 Tissue kit according to the manufacturer’s instructions (Qiagen). Whole-genome shotgun sequencing was performed using the 454 GS FLX-Titanium system (Roche) and MiSeq (Illumina). Reads were assembled using the Newbler assembler version 2.8 (Roche). Primer walking on gap-spanning PCR products from genomic DNA closed the gaps between the assembled sequences. The genome was submitted to RAST (http://rast.nmpdr.org/) for automatic annotation.

The phylogenetic position was elucidated using the 16S rRNA gene sequence. Sequences were aligned using the ARB program (Ludwig *et al.*, 2004), and a phylogenetic tree was reconstructed by the neighbor-joining method using the CLUSTAL_X program (Saitou and Nei, 1987; Thompson *et al.*, 1997).

Absolute differences between charged and polar amino acid residues (CvP bias) and the Ile, Val, Tyr, Trp, Arg, Glu, and Leu amino acid bias (IVYWREL bias) of predicted proteins were calculated according to the methods described by Suhre and Claverie (2003) and Zeldovich *et al.* (2007), respectively. Proteins with less than 2 predicted trans-membrane helices (assessed using TMHMM Server v. 2.0 [Sonnhammer *et al.*, 1998; Krogh *et al.*, 2001]) were used for calculations. We analyzed the G+C content for every 10,000 bases on each genome and the codon usage of amino acids for predicted proteins using the G-language system (Arakawa *et al.*, 2008; 2010).

### Sequence accession numbers

The genome sequence of strain sy52^T^ was deposited in DDBJ/EMBL/GenBank with the accession number AP018712 under the BioProject accession number PRJDB6802 and BioSample accession number SAMD00113976 using DFAST, the DDBJ Fast Annotation and Submission Tool (Tanizawa *et al.*, 2016; 2018). The 16S rRNA gene sequence was also deposited with the accession number LC485113. All DDBJ/EMBL/GenBank accession numbers for analyses are shown in Fig. 3.

## Results

### Growth properties and chemotaxonomic characteristics

**Fig. 1.**
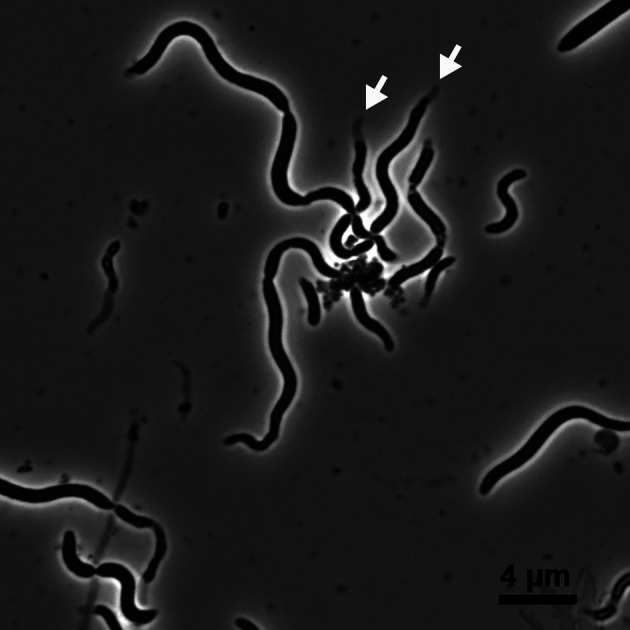
Phase-contrast micrograph of strain sy52^T^. The ‘toga’ structure is indicated by open arrows.

**Fig. 2.**
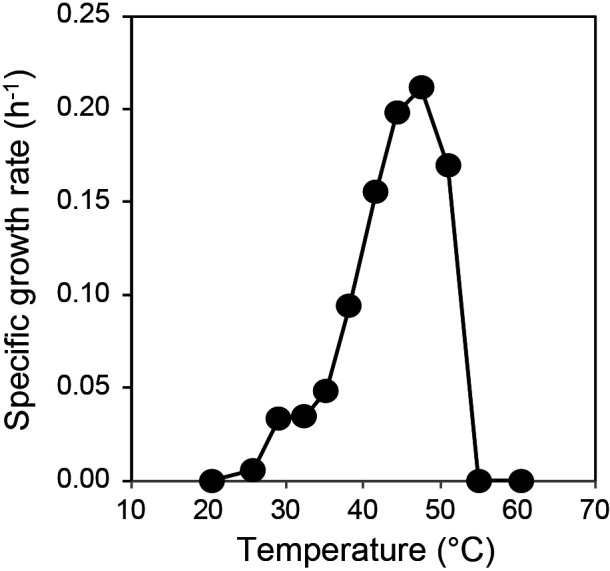
Effects of temperature on the growth of strain sy52^T^.

The cells of strain sy52^T^ had a rod-shaped morphology with the presence of a toga structure, and motility was observed under a microscope. Spiral-shaped cells were detected under optimum growth conditions (Fig. 1). Strain sy52^T^ is a strictly anaerobic bacterium and, thus, was unable to grow under aerobic conditions. It required yeast extract as a growth factor, which was not replaceable by a vitamin mixture. The vitamin solution was not required for its growth. In the presence of 0.2‍ ‍g L^–1^ yeast extract, strain sy52^T^ grew with yeast extract, polypeptone, and starch as energy and carbon sources. However, even in the presence of 0.2‍ ‍g L^–1^ yeast extract, other organic substrates did not stimulate growth. In the presence of yeast extract as energy and carbon sources, strain sy52^T^ used thiosulfate and elemental sulfur as electron acceptors and reduced them to hydrogen sulfide. Growth yield was two-fold higher following their addition than with fermentation. Strain sy52^T^ grew at temperatures ranging between 26 and 51°C, with the optimum temperature being 48°C (Fig. 2). The initial pH range for growth was 5.0–7.0, with an optimum at pH 6.0. The strain grew in 1–5% (w/v) NaCl, with an optimum concentration of 2–4% NaCl. The doubling time under optimum growth conditions was 3 h, and growth yield reached approximately 5×10^7^‍ ‍cells‍ ‍mL^–1^ in the presence of yeast extract and thiosulfate.

The cells of strain sy52^T^ contained C_16:0_ (52% of all fatty acids) as the major fatty acid, and C_16:1_ω7c (13%), C_16:1_ω9c (12%), C_18:1_ω9c (6%), C_18:0_ (6%), C_14:0_ (5%), C_12:0_ (2%), C_18:1_ω7c/ω6c (2%), C_17:1_iso/anteiso (1%), and C_10:0_ (1%) were identified as minor fatty acids.

### Genome sequencing

The complete genome sequence of strain sy52^T^ was elucidated, resulting in a genome that consists of a 2,502,404-bp circular chromosome with a G+C content of 25.8 mol%. Six copies of an rRNA operon and 2,302 predicted protein-coding genes were identified. Six copies of complete rRNA operons obtained from the genome sequence of strain sy52^T^ and their 16S rRNA gene sequences showed slight differences and a similarity of 99.8–100%.

### Phylogenetic position

**Fig. 3.**
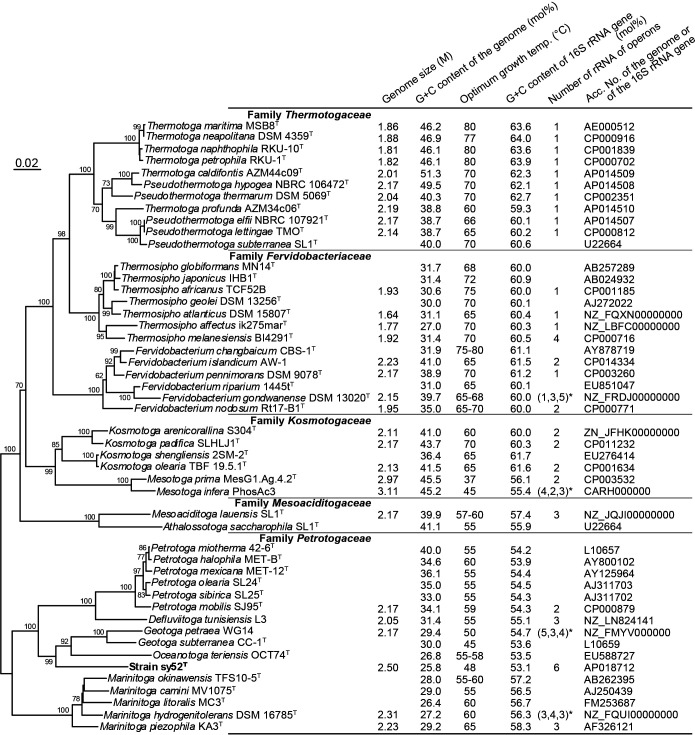
Neighbor-joining phylogenetic tree based on sequences of the 16S rRNA gene and genome size, G+C contents of the genome and 16S rRNA gene, optimum temperature for growth, and the number of ribosomal RNA operons of bacteria of the *Thermotogae* lineage. Bootstrap values are indicated at branch nodes. Genomic G+C contents calculated based on genome sequences are preferentially indicated. DDBJ/EMBL/GenBank accession numbers for analyses are shown in the rightmost column. Bar, 0.02 substitutions per nucleotide position. *They have some partial rRNA operons (numbered 5S, 16S, and 23S): *F. gondwanense* (1, 3, 5); *M. infera*, (4, 2, 3); *G. petraea*, (5, 3, 4); *M. hydrogenitolerans*, (3, 4, 3).

The phylogenetic position of strain sy52^T^ was identified using the 16S rRNA gene sequence. The neighbor-joining tree (Fig. 3) revealed that the strain belonged to the family *Petrotogaceae* in the phylum *Thermotogae*. The 16S rRNA gene sequence of strain sy52^T^ had a similarity of less than 90% with that of species in the phylum *Thermotogae*, and the closest relatives were *Oceanotoga teriensis* (sequence similarity of 87.8%) and *G. subterranea* (87.2%).

### Genome characteristics

**Fig. 4.**
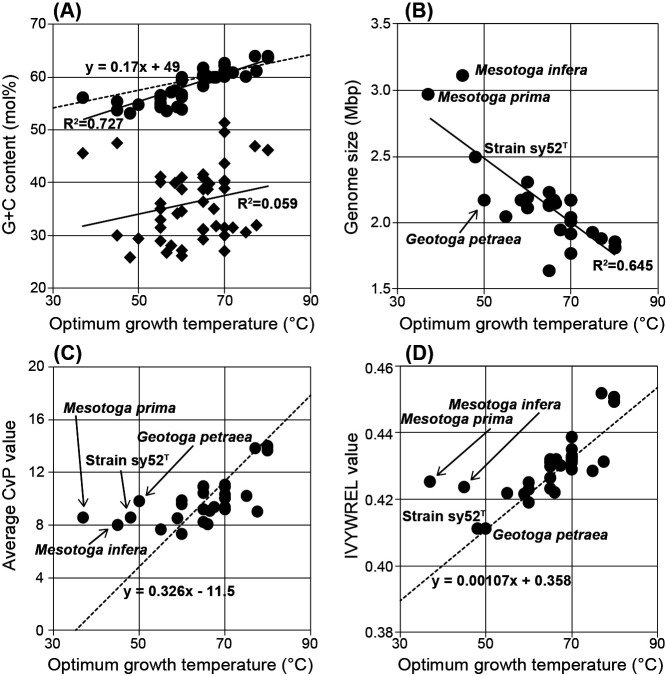
Relationship between the optimum temperature and various parameters of bacteria of the *Thermotogae* lineage: (A) correlations with the G+C content of the 16S rRNA gene (circle) and genome (diamond), the dot-linear mathematical formula based on 406 prokaryotes was from Kimura *et al.* (2006); (B) correlation with genome sizes; (C) correlation with average CvP values, the dot-linear mathematical formula based on 4 bacteria of the *Thermotogae* lineage was from Zhaxybayeva *et al.* (2009); (D) correlation with IVYWREL values, the dot-linear mathematical formula based on 86 prokaryotes denoted by Zeldovich *et al.* (2007).

The G+C contents of the 16S rRNA gene sequences of bacteria of the *Thermotogae* lineage and strain sy52^T^ plotted against their optimum growth temperatures revealed a correlation (Fig. 4A). On the other hand, the G+C content of the whole genome sequence did not correlate with optimum growth temperatures (Fig. 4A). The genome size of strain sy52^T^ was larger than those of the thermophilic and hyperthermophilic species in the *Thermotogae* lineage, but not as large as those of *Mesotoga* species (Fig. 4B). Genome sizes were related to optimum growth temperatures, and species with lower optimum growth temperatures generally had a larger genome. Regarding average CvP values (Fig. 4C), the plots of strain sy52^T^, *M. prima*, *M. infera*, and *G. petraea* were distant from those of species of the *Thermotogae* lineage. The IVYWREL value of strain sy52^T^ against the optimum temperature may harmonize with the linear regression reported by Zeldovich (Zeldovich *et al.*, 2007), whereas those of *Mesotoga* species showed marked deviations (Fig. 4D).

## Discussion

Enrichment and isolation procedures resulted in the successful isolation of strain sy52^T^ from the hydrothermal vent chimney at Suiyo Seamount. According to the phylogenetic analysis based on 16S rRNA gene sequences, the isolated strain belonged to the family *Petrotogaceae* in the phylum *Thermotogae* (Fig. 3). However, sequence similarities with known species were less than 90%, indicating that the strain was phylogenetically independent at the genus level. Characteristics such as the presence of a toga structure (Fig. 1) and energy acquisition by fermentation were similar to bacteria of the *Thermotogae* lineage. On the other hand, strain sy52^T^ grew at temperatures lower than 51°C with an optimum temperature of 48°C (Fig. 2), which is lower than the growth temperature of most bacteria of the *Thermotogae* lineage, with a few exceptions. Based on the optimum growth temperature, it may not be reasonable to state that strain sy52^T^ is a typical mesophile or “mesotoga”; however, it is not a thermophile and displayed better adaptation to a mesophilic environment than other bacteria of the *Thermotogae* lineage. The optimum growth temperature of *G. subterranea* was previously reported to be 45°C (Davey *et al.*, 1993), and, thus, strain sy52^T^ and *G. subterranea* of the family *Petrotogaceae* appear to belong to a lineage that is adapted to mesophilic environments, in contrast to the lineage of the genus *Mesotoga* in the family *Kosmotogaceae*. Therefore, we investigated the relationship between the genome and adaptation to mesophilic environments using genomic information from two lineages.

The genome of strain sy52^T^ is 2.50‍ ‍Mb in length and has a G+C content of 25.8 mol%. The size of its genome was markedly larger than that of other bacteria of the *Thermotogae* lineage (Fig. 3), similar to that observed for genomes of the genus *Mesotoga*, and genome sizes negatively correlated with optimum growth temperatures among bacteria of the *Thermotogae* lineage (Fig. 4B). A relationship may exist between habitat changes and genome sizes; however, adaptations to mesophilic environments only do not indicate habitat changes in the bacterial lineage. The G+C content of strain sy52^T^ was lower than those of any other bacteria of the *Thermotogae* lineage (Fig. 3). This low G+C content may be attributed to the high use of adenine and thymine as the 3rd base of the amino acid code (data not shown). Although a low G+C content occurred in some species, such as those in the family *Petrotogaceae* (Fig. 3), it was not associated with optimum growth temperatures (Fig. 4A). Therefore, the low G+C content of the genome may be due to other factors as well as adaptations to mesophilic environments.

Previous studies proposed an inverse correlation between the G+C contents of 16S rRNA sequences and optimal growth temperatures in bacteria and archaea (Khachane *et al.*, 2005; Kimura *et al.*, 2006; 2007). A similar relationship was observed for bacteria of the *Thermotogae* lineage (Fig. 4A), and the thermodynamic stability of 16S rRNA secondary structures also reflects their habitats. On the other hand, 6 copies of rRNA operons were identified in the genome of strain sy52^T^, and although the possession of multiple rRNA operons may be of significance in the family *Petrotogaceae* (Fig. 3), the number of rRNA operons in strain sy52^T^ is marked compared to others in the phylum. Several divergent/identical 16S rRNA genes were previously shown to be harbored in the genomes of bacteria and archaea, and one base or more dissimilar 16S rRNA genes were detected in almost 50% of genomes (Sun *et al.*, 2013). Although we considered multiple aspects of the possession of a high number of rRNA operons, its significance remains unclear, and its relationship with adaptations to mesophilic environments has not yet been elucidated.

Overrepresentations of charged amino acid residues over polar ones (CvP bias) and IVYWREL amino acids in predicted proteins have been suggested as indicators of the optimal growth temperatures of bacteria and archaea (Suhre and Claverie, 2003; Zeldovich *et al.*, 2007; Taylor and Vaisman, 2010). Previous analyses of some bacteria of the *Thermotogae* lineage revealed that average CvP and IVYWREL values were linearly related to optimal growth temperatures and also that the proteins of *M. prima* were not suitable for a thermophilic environment (Zhaxybayeva *et al.*, 2009; 2012). Although many of the average CvP values are on the line calculated based on four bacteria of the *Thermotogae* lineage reported by Zhaxybayeva *et al.* (2009), the values of strain sy52^T^, *M. prima*, *M. infera*, and *G. petraea* are not on the line (Fig. 4C), indicating that their proteins remain thermophilic. On the other hand, except for two *Mesotoga* species, IVYWREL values were on the line calculated based on 86 bacteria and archaea (Zeldovich *et al.*, 2007). The two indicators of *Mesotoga* species suggest that they still possess thermo-adapted proteins, while proteins of strain sy52^T^ and *G. petraea* adapt to mesophilic environments.

Thermophilic features were previously suggested to be the original characteristics of bacteria of the *Thermotogae* lineage (Zhaxybayeva *et al.*, 2009), and based on the amino acids in predicted proteins, strain sy52^T^ and *G. petraea* showed better adaptation to a mesophilic environment than any other known species in the phylum *Thermotogae*. In mesophilic species in the phylum *Thermotogae*, the essential factor for growth temperature is the G+C content of the 16S rRNA sequence rather than its amino acid composition. In addition, we focused on the extremely low genomic G+C content and high number of rRNA operons in strain sy52^T^; however, it is pure speculation that they are traits of adaptation to a different environment. More isolates of “mesotoga” and further details of their genomic characteristics are needed to completely clarify adaptations to mesophilic environments by the phylum *Thermotogae*.

A phylogenetic analysis based on 16S rRNA gene sequences (Fig. 3) revealed that strain sy52^T^ belonged to the family *Petrotogaceae*, and the characteristics of strain sy52^T^, such as it being a moderate thermophile, requiring NaCl for growth, possessing C_16:0_ as a major cellular fatty acid, and having a low genomic G+C content, were similar to those of genera in the family (Table 1). However, sequence similarities between the strain and species in the family were less than 90%, and the difference was sufficient to denote a new genus for the strain (Stackebrandt and Goebel, 1994). Based on physiological and phylogenetic evidence, a novel taxon, *Tepiditoga spiralis* gen. nov., sp. nov., belonging to the family *Petrotogaceae*, is proposed.

### Description of *Tepiditoga* gen. nov.

*Tepiditoga* (Te.pi.di.to’ga. L. adj. *tepidus* moderately warm; L. fem. n. *toga* outer garment; N.L. fem. n. *Tepiditoga* a moderately warm garment).

Cells are rods with a sheath-like outer structure. Spiral rod-shaped cells are observed under optimum growth conditions. It is obligately anaerobic and chemoorganotrophic. It is moderately thermophilic and moderately halophilic. It grows by fermentation and reduces sulfur compounds. The major cellular fatty acid is C_16:0_. Its phylogenetic position based on the 16S rRNA gene sequence is in the family *Petrotogaceae*. The type species is *Tepiditoga spiralis*.

### Description of *Tepiditoga spiralis* sp. nov.

*Tepiditoga spiralis* (spi.ra’lis. L. adj. *spiralis* spiral).

It has the following characteristics in addition to those given in the genus description. Under optimum growth conditions, cells are spiral rod-shaped and motile. It reduces thiosulfate and elemental sulfur to sulfide. Yeast extract, polypeptone, and starch are used as growth substrates. It does not grow on a sole substrate and yeast extract is necessary for growth. It grows at temperatures ranging between 26 and 51°C, with optimal growth at 48°C. The initial pH for growth is pH 5.0–7.0, with an optimum at pH 6.0. The NaCl concentration for growth ranges between 1 and 5% (w/v), with an optimum at 2–4%. The predominant cellular fatty acid is C_16:0_. C_16:1_ω7c, C_16:1_ω9c, C_18:1_ω9c, C_18:0_, C_14:0_, C_12:0_, C_18:1_ω7c/ω6c, C_17:1_iso/anteiso, and C_10:0_ are minor fatty acids.

The type strain, sy52^T^ (=NBRC 112788^T^=DSM 105848^T^), was isolated from a hydrothermal chimney in Suiyo Seamount, the Izu-Bornin Arc, the western Pacific Ocean. The genomic G+C content of the type strain is 25.8 mol%.

## Citation

Mori, K., Sakurai, K., Hosoyama, A., Kakegawa, T., and Hanada, S. (2020) Vestiges of Adaptation to the Mesophilic Environment in the Genome of *Tepiditoga spiralis* gen. nov., sp. nov.. *Microbes Environ ***35**: ME20046.

https://doi.org/10.1264/jsme2.ME20046

## Figures and Tables

**Table 1.** Characteristics of strain sy52^T^ and genera in the family *Petrotogaceae*.

Characteristics	strain sy52^T^	*Petrotoga*	*Defluviitoga*	*Geotoga*	*Oceanotoga*	*Marinitoga*
Optima for growth						
temperature (°C)	48	55–60	55	45–50	55–58	55–65
pH	6.0	6.5–8.0	6.9	6.5	7.3–7.8	5.5–7.0
NaCl (w/v [%])	2–4	1–6	0.5	3	4–4.5	2–4
Growth temperature range (°C)	26–51	30–65	37–65	30–60	25–70	25–70
Reduction of sulfur compounds	+	+/–	+	+	+	+
Major fatty acids	C_16:0_	C_16:0_, C_18:1_	C_16:0_, C_18:1_ω9c	C_16:0_, C_16:1_	C_16:0_	C_16:0_, C_18:0_
Genomic G+C content (mol%)	25.8	33.0–40.0	31.4	29.4–30.0	26.8	26.4–29.2
Reference	this study	(Davey *et al.*, 1993; Miranda-Tello *et al.*, 2007)	(Ben Hania *et al.*, 2012)	(Davey *et al.*, 1993)	(Jayasinghearachchi and Lal, 2011)	(Nunoura *et al.*, 2007; Postec *et al.*, 2010)

+, positive; –, negative.
